# Etiologic Agents and Diseases Found Associated with Clinical Aspergillosis in Falcons

**DOI:** 10.1155/2011/176963

**Published:** 2011-06-07

**Authors:** Walter Tarello

**Affiliations:** Avian & Exotic Division, Pet Connection Veterinary Clinic, P.O. Box 450288, Dubai, UAE

## Abstract

The aim of this study was to describe parasitological, microbiological, and pathological findings associated with the isolation of *Aspergillus* species in 94 clinically diseased captive falcons from Dubai. Concomitant agents and/or diseases were identified in 64 cases, causing either single (*n* = 36) or multiple coinfections (*n* = 28). Diagnoses found more often in association with aspergillosis were chronic fatigue and immune dysfunction syndrome (CFIDS) (*n* = 29), *Caryospora* sp. (*n* = 16), *Serratospiculum seurati* infestation (*n* = 14), cestodiasis (*n* = 6), bumblefoot (*n* = 5), trematodosis due to *Strigea falconispalumbi* (*n* = 5), trichomoniasis (*n* = 4), *Babesia shortti* (*n* = 4), *Mannheimia (Pastorella) haemolytica* (*n* = 4), interstitial hepatitis (*n* = 4), *Escherichia coli* (*n* = 3), and *Clostridium perfringens* enterotoxemia (*n* = 2). Compared with a control group of 2000 diseased falcons without evidence of aspergillosis, the prevalence of *Babesia shortti*, CFIDS, *Mannheimia (Pastorella) haemolytica, Escherichia coli*, and falcon herpes virus infection was conspicuously higher in association with aspergillosis. These entities may be considered suitable candidates as predisposing factors for the mycosis.

## 1. Introduction

Aspergillosis is considered the most common systemic mycosis in birds [1] and the most important cause of death in captive falcons [2, 3]. Infections with *Aspergillus fumigatus* and, less commonly, with *A. flavus*, *A. terreus, *and *A. niger* apparently share the same clinical importance in raptors held in captivity [4]. Clinical signs in birds are nonspecific and include reduction in appetite, weight loss, dyspnoea, lethargy, and death [5]. 

Avian aspergillosis involves mainly the lower respiratory tract [6]. This also occurs in falcons, in which the majority of reported cases are isolated from the air sacs [4]. These fungi are ubiquitous, but they become pathogenic mainly under stressful conditions, producing opportunistic infections as a result of inhalation of *Aspergillus* spores coupled with compromised immune functions in the host or in association with prolonged diseases [6–8]. Poor ventilation, malnutrition, toxins, vaccinations, long-term use of antibiotics and corticosteroids, hot-humid climate, and stress-associated conditions, such as recent capture, training, and change of ownership, are frequently mentioned as environmental precipitating factors influencing the onset and duration of aspergillosis in falcons [7–10]. 

Unfortunately, what actually causes immunosuppression and/or prolonged disease is rarely mentioned in the literature, because dual or multiple infections with potentially immunocompromising or chronically persistent pathogens (i.e., *Mycobacterium* spp., *Babesia shortti*, and *Serratospiculum* sp. nematode) are only sporadically recorded in raptors [11, 12] and other birds with aspergillosis [8, 13]. It is not clear whether these infections are truly rare, underdiagnosed, diagnosed but underestimated, or simply rarely reported. 

Thus, it seemed important to assess to what extent the involvement of concurrent agents occurs in the condition. This study provides a list of the pathogens recorded recently in 94 captive falcons from Dubai with a definitive diagnosis of aspergillosis and compares their prevalence with a control group of 2000 randomly chosen diseased falcons without clinical, biochemical, and endoscopic evidence of aspergillosis.

## 2. Materials and Methods

The study was carried out at the Al Wasl Veterinary Clinic, Dubai, United Arab Emirates, from August 2005 to October 2006. Biopsy samples were collected during endoscopy from the air sacs of 94 diseased falcons diagnosed with fungal disease of the lower respiratory tract and showing compatible clinical signs. There were 30 gyrfalcons (*Falco rusticolus*), 19 saker falcons (*Falco cherrug*), 12 peregrine falcons (*Falco peregrinus*), 2 Barbary falcons (*Falco pelegrinoides*), and 31 hybrid falcons. Specimens were cultured in Sabouraud's chloramphenicol agar (bioMerieux) and incubated at 37°C for three to five days. Ninety-six fungal isolates were collected from 94 falcons. The species involved were identified, on the basis of macroscopic appearance of cultures and their microscopic morphological features, after staining with lactophenol aniline, as follow: *Aspergillus fumigatus* (57 cases), *A*. *flavus* (13 cases), *A*. *terreus* (12 cases), *A*. *niger* (10 cases), *A*. *niger* + *A*. *flavus* (1 case), and *A*. *niger* + *A*. *fumigatus* (1 case). Information evaluated included signalment, duration of the disease, clinical signs, concurrent diseases, and previous diagnosis and treatments. 

Treatment for aspergillosis was based on antifungal medicaments, such as amphotericin B, itraconazole, and voriconazole. 

Blood samples of 1.5 mL were collected from the medial metatarsal vein and used for the preparation of Wright-stained blood smears and for routine biochemical and hematological tests. Microscopic search of parasite eggs was performed on fresh feces. Samples for bacteriology and virology tests were collected with sterile swabs from crop, trachea, air sacs, and cloaca in selected cases and sent to the Central Veterinary Research Laboratory (CVRL) of Dubai. In some cases, kidney and liver biopsies as well as carcasses were also sent to the CVRL for histopathology and postmortem examination. The prevalence of concurrent diseases and/or agents was compared with those of a control group of 2000 randomly chosen diseased falcons without clinical, biochemical, and endoscopic evidence of aspergillosis examined in the Al Wasl Veterinary Clinic, Dubai, during the same period.

## 3. Results

Concomitant agents and/or diseases were identified in 64 cases, causing either single (*n* = 36) or multiple coinfections (*n* = 28), and these are listed in Table 1. Death occurred in 16 cases (Table 1). 

The most commonly associated parasitic diseases were coccidiosis due to *Caryospora* spp. infection (*n* = 16) and *Serratospiculum seurati* (*n* = 14). Infestations with the trematode *Strigea falconispalumbi* (*n* = 5) and *Capillaria* spp. (*n* = 1) were found only associated with *A*. *flavus* infection, as well as one fatal case of leucocytozoonosis. 

Trichomoniasis (*n* = 4) was mostly associated with *A*. *fumigatus* (*n* = 3). Viral diseases identified in the study group were falcon herpes virus (*n* = 2) and pox virus (*n* = 2) infection. 

 The most commonly diagnosed bacterial diseases were the chronic fatigue and immune dysfunction syndrome (CFIDS) (*n* = 29) [14], bumblefoot or pododermatitis (*n* = 5), *Mannheimia* (*Pastorella*) *haemolytica* (*n* = 4), *Escherichia coli* (*n* = 3), and *Clostridium perfringens* enterotoxemia (*n* = 2). Concurrent CFIDS and bumblefoot were diagnosed in 2 out of five cases of pododermatitis associated with aspergillosis, apparently confirming the link previously noted between the two conditions [15]. 

Fatal outcomes were mostly seen associated with CFIDS (*n* = 4), pastorellosis (*n* = 2), herpes virus infection (*n* = 2), and amyloidosis (*n* = 2). Two fatal cases of *Aspergillus fumigatus* infection were associated with CFIDS and *in vivo* resistance to long-term treatment with voriconazole. 

Comparison between prevalence of concurrent diseases and/or agents found in diseased falcons with (*n* = 94) and without aspergillosis (*n* = 2000) shows that *Babesia shortti*, CFIDS, *Mannheimia haemolytica*, *Escherichia coli,* and falcon herpes virus infection are significantly more common in birds with aspergillosis (Table 2). 

## 4. Discussion

*Aspergillus fumigatus* is one of the major clinical isolates in falcons with aspergillosis [16]. Results reported here confirm such prevalence, because 58 (60%) of the recorded cases were due to *A*. *fumigatus*. Infections with *A*. *flavus* (*n* = 14), *A*. *terreus* (*n* = 12), and *A*. *niger* (*n* = 12) were also common. In this study, concomitant primary pathogenic agents and/or diseases were identified in approximately 2/3 of the *Aspergillus*-positive falcon cases (Table 1), 1/3 of which showed multiple coinfections, thus apparently confirming the claimed opportunistic role of aspergillosis. 

However, in most cases, this assumption proved incorrect when the prevalence rates of the etiologic agents involved were compared with those reported in a large group of diseased falcons (*n* = 2000) without aspergillosis. In fact, the recorded rates of *Caryospora* spp., *Serratospiculum seurati*, *Strigea falconispalumbi*, candidosis, capillariasis, leucocytozoonosis, bumblefoot, *Clostridium perfringens* entero-toxaemia, and pox virus infection do not substantially differ between the 2 groups (Table 2). Furthermore, and curiously, cestodiasis and trichomoniasis are significantly less prevalent in falcons with aspergillosis (Table 2). 

Therefore, these agents do not constitute a risk factor in the development of aspergillosis in falcons, as well as other bacteria less commonly encountered such as *Klebsiella ozanonae* (*n* = 1), *Moraxella* sp. (*n* = 1), *Staphylococcus aureus* (*n* = 1), and *Acinetobacter baumannii* (*n* = 1), occasionally associated with aspergillosis (Table 1). Candidosis was diagnosed once in association with *A*. *fumigatus* infection. This is one of the most common fungal diseases in birds [17]. The low prevalence recorded in this study seems at least unusual, and it is probably due to underdiagnosis. Previous association between Candida and *Aspergillus* has been described in 2 Amazon parakeets [13]. 

Why *Babesia shortti*, CFIDS, *Mannheimia* (*Pastorella*) *haemolytica*, *Escherichia coli,* and falcon herpes virus infections were notably more common in birds with aspergillosis (Table 2)? Do they really constitute predisposing factors for the mycotic disease or are they simply the result of mistaken overdiagnosis? 

We should remember first that in human medicine, underlying diseases favouring *Aspergillus* are relatively well known and are the followings: hematological malignancies (33%), chronic obstructive pulmonary disease (22%), bone marrow transplant (14%), HIV infection (11%), and absolute neutropenia (10%) [18]. Hematological malignancies are rarely described in falcons and not in association with aspergillosis [19]. With the exclusion of cancer, it is acknowledged in veterinary medicine that aspergillosis generally occurs mainly in immune-compromised hosts [10]. 

Babesiosis is recognized as an immune-compromising disease [20]. In this study, *Babesia shortti* was diagnosed in 4 chronically ill falcons [21] with aspergillosis (1 *A*. *fumigatus*, 2 *A*. *flavus,* and 1 *A*. *niger*). Clinical manifestation of babesiosis varies from subclinical signs in apparently healthy animals with low parasitaemia to severe disease associated with high parasitaemia, showing weight loss, anorexia, lethargy, vomiting, seizure, and blood in the stool [21]. These signs are nonspecific and overlapping those of aspergillosis in most cases. Recently, the first molecular characterization of the organisms revealed that *Babesia shortti* is closely related (97%) to *Babesia poelea*, recently described in Brown boobies (*Sula leucogaster*), and that it belongs to the clade of piroplasms previously detected in humans, dogs, and wild ungulates in the western United States [22]. Canine babesiosis is known to cause immune dysfunctions which favour secondary opportunistic infections [23, 24]. Previous report of concomitant occurrence of *B*. *shortti* and *A*. *fumigatus* leading to fatal outcomes [12] agrees with the hypothesis supported here that babesiosis tends to predispose and exacerbate the mycotic disease. 

In this study, the isolation of herpes virus in two fatal cases of falcon hepatitis with concomitant aspergillosis (*A*. *fumigatus* and *A*. *terreus*) is probably the first occurrence of such association recorded in veterinary literature. Identification of the causative virus was done postmortem. It is not excluded that more cases of herpes virus infection might have been missed, lacking a specific suspect and/or fatal outcomes. 

*Mannheimia* (*Pastorella*) *haemolytica*, a Gram-negative highly pathogenic bacterium causative agent of the pneumonic pastorellosis, was associated with 4 cases of aspergillosis in this study (1 *A*. *fumigatus*, 2 *A*. *flavus,* and 1 *A*. *niger*), 2 of which showing fatal outcomes despite aggressive antifungal therapy. Table 2 shows that *M*. *haemolytica* was strikingly more prevalent in falcons with aspergillosis (4.25%) when compared with the control group (0.1%) apparently indicating a predisposing action for the mycotic disease. Clinical signs of avian pastorellosis, such as general malaise, respiratory distress, and diarrhea [25], are vague and partially overlapping those due to aspergillosis. Lack of recognition and of preventive treatment for underlying pastorellosis may lead to poor prognosis and negative therapy outcomes for the concomitant aspergillosis. The same should apply to *Escherichia coli* (*n* = 3, 1 death) (Table 1), which was prevalently found associated with aspergillosis (3.12% versus 1%) in this study (Table 2). These bacteria are potentially pathogenic for falcons [25], causing chronic diseases and immune dysfunctions that can predispose to aspergillosis and complicate its therapy. *Aspergillus* sp. association with *Pastorella multocida* has been previously described in turkeys [1].

In human medicine, aspergillosis also occurs as an opportunistic infection in the condition named idiopatic CD4+ T lymphocytopenia [26, 27], a subtype of the chronic fatigue syndrome (CFS), also called chronic fatigue and immune dysfunction syndrome (CFIDS) due to the frequency with which autoimmune defects and cellular and humoral deficiencies are recorded in human and animal patients as well [28]. It is acknowledged that about 1/10 of human CFIDS patients shift naturally to the condition called idiopathic CD4+ T cell lymphocytopenia (ICL), which is characterized by decreased CD4+ T cells count in the absence of HIV infection and occasional association with leukopenia and pan-hypogammaglobulinemia. Most cases of ICL, also called HIV-negative AIDS, fulfil the CDC criteria for CFIDS, and the two conditions appear today as variations in severity of a single disease [28]. Immunological anomalies such as leukopenia, lymphopenia, and hypogammaglobulinemia have been seen in birds of prey [14] and dogs and cats previously diagnosed with CFIDS [28]. It is comparatively interesting to note that CFIDS in this study was the underlying disease most commonly diagnosed in captive falcons with proved aspergillosis (29 reported cases and 4 deaths). In my experience, CFIDS is associated with staphylococcal infection and bacteraemia in birds of prey [14] and other animals [28–34]. The prevalence reported here should not be controversial because the prevalence of *Staphylococcus* spp. infections in the Spanish imperial eagle (*Aquila adalberti*) was as high as 45% in chicks handled without gloves, and 4% in chicks handled with gloves [35]. Apparently, the human-animal contact was the way of transmission. Reported captive falcons diagnosed with CFIDS are routinely subjected to human-animal interaction, intense training, and, therefore, oxidative stress, that favour CFIDS [31].

Conditioned illnesses are difficult to treat when underlying primary agents are not preliminarily eliminated [24]. As a confirmation, two fatal cases of *A*. *fumigatus* infection associated with CFIDS showed *in vivo* resistance to voriconazole, currently considered the best antifungal agent available on the market [4]. 

CFIDS was diagnosed on the basis of the presence of micrococci in the circulating blood, as detected on Wright-stained blood smears by light microscopy, high creatine kinase levels, and suggestive clinical signs, such as weight loss, lethargy, poor appetite, and reduced speed and strength in flight [14, 29]. The micrococcal forms observed in the blood were indistinguishable from those previously seen in birds of prey diagnosed with CFIDS [14]. The condition, also called chronic fatigue syndrome (CFS), can affect a variety of species, including humans [30–34] and falcons [29]. In this author experience, detection of micrococci in blood smears seems to be a diagnostic criterion for CFIDS diagnosis in many species [30–34], being the most remarkable haematological difference between chronically fatigued and healthy patients and by revealing the association with an underlying *Staphylococcus* spp. bacteraemia [28]. Blood cultures are less sensitive [30–34] due to a wide range of factors affecting the growth of bacteria from blood samples, including plate's contamination. Therefore, blood cultures were not performed in the present case series. In a number of animal and human CFS cases reported till now, blood cultures proved nonetheless positive for the growth of strains of *Staphylococcus intermedius*, *S*. *xylosus*, *S*. *epidermidis*, *St*. *lugdunensis*, *S*. *cohnii*, *S*. *warneri,* and *S*. *chromogenes* [30–34] confirming that the micrococci seen in the blood smears actually were coagulase-negative staphylococci invading the bloodstream. 

Today, these staphylococci are not considered benign as previously thought, since they can also cause meningoencephalitis in dogs and human beings [36]. Toxin-secreting staphylococci produce a group of proteins called superantigens, which are toxins capable of inducing a polyclonal T-cell activation and high interleukin-2 secretion. In the long run, this mechanism may induce autoimmunity, stimulating the growth of a subset of lymphocytes that recognize the “self” as a stranger. Such nonspecific activation can also lead to immune deficiency because certain T-cells subpopulations vigorously proliferate and finally die, leaving an open door to opportunistic agents [28], such as *Aspergillus* species. 

CFIDS is not widely known to occur in animals and consequently was not considered, up to now, a possible concurrent and/or favoring agent for aspergillosis. In human medicine, CFIDS is perceived as underdiagnosed [31]. The general lack of CFIDS diagnosis in veterinary medicine should not be regarded as evidence of absence and for excluding CFIDS as a true predisposing factor for aspergillosis, considering that these bacteria are difficult to isolate and their presence in the host is not obvious [36]. Antifungal therapy is usually unsuccessful in chronic, advanced cases of aspergillosis [6] suggesting that an early therapy for aspergillosis (and for concurrent agents) would be appropriate in most cases. It is acknowledged that in conditioned illnesses the eradication of concurrent pathogens is a prerequisite for an effective therapy [24]. Little information is available about the effectiveness of antifungal drugs in multiple infections. Resistance to voriconazole and itraconazole has been reported in *Aspergillus* species in vitro, and decreased susceptibility has been noticed recently in *Aspergillus* sp. to multiple antifungal agents [37]. If this is linked to the presence of concurrent pathogens, it is hard to say at present time and should be further evaluated. Aspergillosis seems uncommon in the wild [38]. On the contrary, it is common in captive and domesticated conditions [5], indicating that environmental factors, including human manipulation ensuing potential transmission of concurrent favoring pathogens, also play their role. To which extent external factors influence the course of aspergillosis in falcons is difficult to determine quantitatively. Minimizing stress and nutritional factors does not eliminate aspergillosis when it is established. Recovery of potentially synergic or feasibly immunosuppressive agents in falcons with aspergillosis should be considered a step toward a better understanding of the pathogenesis of the mycosis. 

## Figures and Tables

**Table 1:** Etiologic agents and diseases found associated with aspergillosis in falcons.

Concurrent diseases		*Aspergillus* isolates				
	*A*. *fumigatus *	*A*. *flavus *	*A*. *terreus *	*A*. *niger *	Total	Deaths
	58 isolates	14 isolates	12 isolates	12 isolates	96	16
*Parasites *						
*Caryospora* spp.	7	4	1	4	16	—
*Serratospiculum* spp.	7	1	1	5	14	1
Cestodes	1	1	1	3	6	—
Trematodes (*S*. *falconis*)	—	5	—	—	5	1
Trichomoniasis	3	1	—	—	4	—
*Babesia* *shortti *	1	2	—	1	4	—
Candidosis	1	—	—	—	1	—
*Capillaria* spp.	—	1	—	—	1	—
*Leucocytozoon* *toddi *	—	1	—	—	1	1

*Bacteria *						
(CFIDS)	17	4	6	2	29	4
Bumblefoot	3	1	—	1	5	—
*Mannheimia* *haemolytica *	1	2	—	1	4	2
*Escherichia* *coli *	1	1	—	1	3	1
*Clostridium* *perfringens *	1	—	1	—	2	1
*Klebsiella* *ozanonae *	1	—	—	—	1	—
*Moraxella* sp.	—	1	—	—	1	—
*Staphylococcus* *aureus *	—	—	1	—	1	—
*Acinetobacter* *baumannii *	—	—	—	1	1	—
Ehrlichiosis	0	1	0	0	1	—

*Virus*						
Falcon herpes virus	1	—	1	—	2	2
Pox virus	1	—	—	1	2	1

*Other diseases *						
Interstitial hepatitis	2	2	—	—	4	—
Amyloidosis	1	—	—	1	2	2
Chronic nephritis	—	—	1	—	1	—

**Table 2:** Comparison between prevalences of aetiologic agents and diseases found in falcons with and without aspergillosis.

Agent/Disease	With aspergillosis			Without aspergillosis	
	(*n* = 94)	%		(*n* = 2000)	%
*Parasites *					
*Caryospora* spp.	(16)	17.00	~	(317)	15.80
*Serratospiculum* *seurati *	(14)	14.90	~	(253)	12.65
Cestodes	(6)	6.38	<	(163)	8.15
Trematodes	(5)	5.32	~	(101)	5.05
Trichomoniasis	(4)	4.25	<	(148)	7.40
*Babesia* *shortti *	(4)	4.25	>	(22)	1.10
Candidosis	(1)	1.06	~	(13)	0.65
*Capillaria* spp.	(1)	1.06	~	(31)	1.55
*Leucocytozoon* *toddi *	(1)	1.06	~	(16)	0.80

*Bacteria*					
CFIDS	(29)	30.85	>	(240)	12.00
Bumblefoot	(5)	5.32	~	(81)	4.05
*Mannheimia* *haemolytica *	(4)	4.25	>	(2)	0.10
*Escherichia* *coli *	(3)	3.12	>	(20)	1.00
*Clostridium* *perfringens *	(2)	2.13	~	(29)	1.45

* Viruses *					
Falcon herpes virus	(2)	2.13	>	(6)	0.30
Pox virus	(2)	2.13	~	(44)	2.20
